# Giant spin hydrodynamic generation in laminar flow

**DOI:** 10.1038/s41467-020-16753-0

**Published:** 2020-06-15

**Authors:** R. Takahashi, H. Chudo, M. Matsuo, K. Harii, Y. Ohnuma, S. Maekawa, E. Saitoh

**Affiliations:** 1 Natural Science Division, Faculty of Core Research, Ochanomizu University, Otsuka, Bunkyo-ku, Tokyo 112-8610 Japan; 2 Advanced Science Research Center, Japan Atomic Energy Agency, Tokai, 319-1195 Japan; 3 Spin Quantum Rectification Project, ERATO, Japan Science and Technology Agency, Sendai, 980-8577 Japan; 4 Advanced Institute for Material Research, Tohoku University, Sendai, 980-8577 Japan; 5 Kavli Institute for Theoretical Sciences, University of Chinese Academy of Sciences, Beijing, 100190 China; 6 CAS Center for Excellence in Topological Quantum Computation, University of Chinese Academy of Sciences, Beijing, 100190 China; 7 Riken Center for Emergent Matter Science (CEMS), Wako, 351-0198 Japan; 8 Department of Advanced Functional Materials Research, National Institutes for Quantum and Radiological Science and Technology, Takasaki, Gunma 370-1292 Japan; 9 Institute for Materials Research, Tohoku University, Sendai, 980-8577 Japan; 10 Department of Applied Physics, The University of Tokyo, Hongo, Bunkyo-ku, Tokyo 113-8656 Japan

**Keywords:** Spintronics, Fluid dynamics

## Abstract

Hydrodynamic motion can generate a flux of electron-spin’s angular momentum via the coupling between fluid rotation and electron spins. Such hydrodynamic generation, called spin hydrodynamic generation (SHDG), has recently attracted attention in a wide range of fields, especially in spintronics. Spintronics deals with spin-mediated interconversion taking place on a micro or nano scale because of the spin-diffusion length scale. To be fully incorporated into the interconversion, SHDG physics should also be established in such a minute scale, where most fluids exhibit a laminar flow. Here, we report electric voltage generation due to the SHDG in a laminar flow of a liquid-metal mercury. The experimental results show a scaling rule unique to the laminar-flow SHDG. Furthermore, its energy conversion efficiency turns out to be about 10^5^ greater than of the turbulent one. Our findings reveal that the laminar-flow SHDG is suitable to downsizing and to extend the coverage of fluid spintronics.

## Abstract (web summary)

In spin hydrodynamic generation originating from the coupling of mechanical rotation in a fluid and electron spin, fluid vorticity can be converted into an electric voltage via a spin current. Here, the authors demonstrate experimentally that the energy conversion in a laminar flow regime is strongly enhanced over the turbulent regime.

## Introduction

A spin current, a flow of spin angular momentum, has enabled the interconversion among various kinds of physical entities such as electricity^1,2^, magnetization^1,2^, heat^2–6^, and mechanical motion like elastic motion^7–11^ and liquid motion^12,13^. Such spin-mediated interconversion is realized on a micro or nano scale^1,2,14^ and has been extensively studied in the field of spintronics. In this stream, recent study has revealed that fluid mechanical motion can also act as a constituent of this interconversion framework: spin hydrodynamic generation (SHDG). The SHDG has attracted much attention not only in the field of spintronics^7,8,14–18^ but also in a wide range of fields including nuclear physics^19^.

**Fig. 1:**
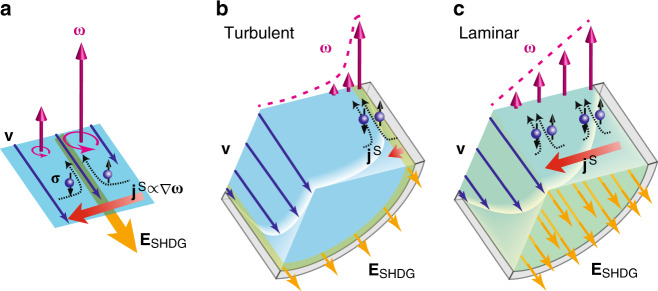
**Spin hydrodynamic generation in a cylindrical channel**. **a** A schematic illustration of SHDG. **v**, ***ω***, ***σ***, **j**^S^, and **E**_SHDG_ are the flow velocity, the vorticity, the spin polarization, the spin current caused by SHDG and the electromotive force caused by the inverse spin Hall effect in a liquid metal flow, respectively. **b**, **c** Geometries for SHDG in a turbulent flow (**b**) and in a laminar flow (**c**). Each illustration represents a cut-out part of a fluid flow in a cylindrical channel where SHDG is induced.

The origin of SHDG is the spin-rotation coupling, that is, the coupling between electron spin and local mechanical rotation of a fluid, or the vorticity, as shown in Fig. 1a. The driving force for the spin current induced by SHDG should strongly depend on vorticity distribution in a fluid flow. Therefore, SHDG is categorized reflecting the two typical regimes of fluid dynamics, that is, turbulent flow and laminar flow regimes. In a turbulent flow in a cylindrical channel (Fig. 1b), electromotive force should be generated only near the inner wall to give rise to an edge charge current. In a laminar flow (Fig. 1c), on the other hand, it is generated all over the cross section of a channel to give rise to a bulk one, where giant SHDG is expected. In micro or nano scale devices, a fluid flow usually becomes laminar^20^. Therefore, to make the most of SHDG in such devices, it is necessary to establish SHDG in laminar flow states.

The mechanism of SHDG is as follows. When a liquid metal flows in a cylindrical channel, vorticity ***ω*** = ∇ × **v** is induced with spatial distribution shown in Fig. 1a, where **v** is the fluid velocity. In the presence of the spin-rotation coupling in a liquid metal, the vorticity gives rise to driving force for conduction-electron spin current^12,13^, and the gradient of the driving force causes a spin current,

1 $${{\mathbf{j}}}^{{\mathrm{S}}}=-\frac{{\sigma }_{0}\hbar }{4{e}^{2}}\nabla \delta {\boldsymbol{\mu }}\simeq -{\lambda }^{2}\xi \nabla {\boldsymbol{\omega }},$$

where **j**^S^ is a spin current, *δ****μ*** is the driving potential for the spin current (the vector direction means the polarized direction ***σ***), *λ* is the spin-diffusion length, and *σ*_0_ is the electric conductivity. *ξ* is the viscosity correction to the Newtonian fluid originating from the angular momentum conversion between the fluid vorticity and the electron spin due to SHDG^12,13^. The equation predicts that a spin current flows along the spatial gradient of ***ω*** with its polarized vector lying in the ***ω*** direction. The spin current generated in the liquid metal is then converted into an electric field, **E**_SHDG_, in the fluid flow direction via the inverse spin Hall effect in the liquid metal.

In a turbulent flow state (Fig. 1b), where SHDG has been first reported^12^, the vorticity gradient is located near the inner wall, meaning that the generation of **E**_SHDG_ is limited near the inner wall. The area where SHDG occurs was estimated to be about 18% in a cross section of the fluid channel, and thus the rest area of the cross section does not contribute the generation but rather reduces the measured voltage due to short circuit currents. In this estimation, we considered one of the turbulent cases^12^, where a liquid Hg flows with the mean flow velocity of 2.1 m s^−1^ in a fluid channel whose inner diameter is 400 μm. The estimated area was determined as a cross section between the end of the viscous sublayer and the positions where  ∇***ω*** becomes one-tenth less than that at the end of the viscous sublayer, at which  ∇***ω*** is maximized. Here, the magnitude of  ∇***ω*** is proportional to ${\left(r-{r}_{0}\right)}^{-2}{\,}$^21^, where *r*_0_ is the inner radius and *r* represents the radial position whose origin is at the center of the channel. This distribution stems from spatial distribution of **v**. In a turbulent flow state, **v** is almost constant in a channel but abruptly changes to zero very close to the inner wall, because momentum transfer perpendicular to the flow direction, origin of the turbulence, is suppressed due to the viscosity near the inner wall.

The localization of the generation is a major drawback of SHDG in turbulent flows from the viewpoint of energy efficiency. In a laminar flow (Fig. 1c), on the other hand, the spatial distribution of **v** pervades in a parabolic way, and thus ***ω*** gradient is created all over the cross section of a channel. According to the spatial distribution of **v** in a laminar flow (the Hagen–Poiseuille flow^21^), the magnitude of  ∇***ω*** is independent of *r*, meaning that **E**_SHDG_ in a laminar flow can be generated all over the cross section of a fluid channel. This implies that SHDG can be much more efficient in a laminar flow than that in a turbulent flow. Therefore, SHDG in a laminar flow can be not only suitable for spin-driven micro or nano devices but also beneficial in the viewpoint of the energy conversion efficiency.

Here we show SHDG phenomenon in a laminar flow regime. In the present study, the laminar-flow SHDG is found to obey a unique scaling rule and continuously change to the turbulent one across a transition flow regime. Our major finding in the present study is that the energy conversion efficiency of the SHDG is exceedingly enhanced in the laminar flow regime.

## Results

### Description of fluid channel for SHDG measurement

**Fig. 2:**
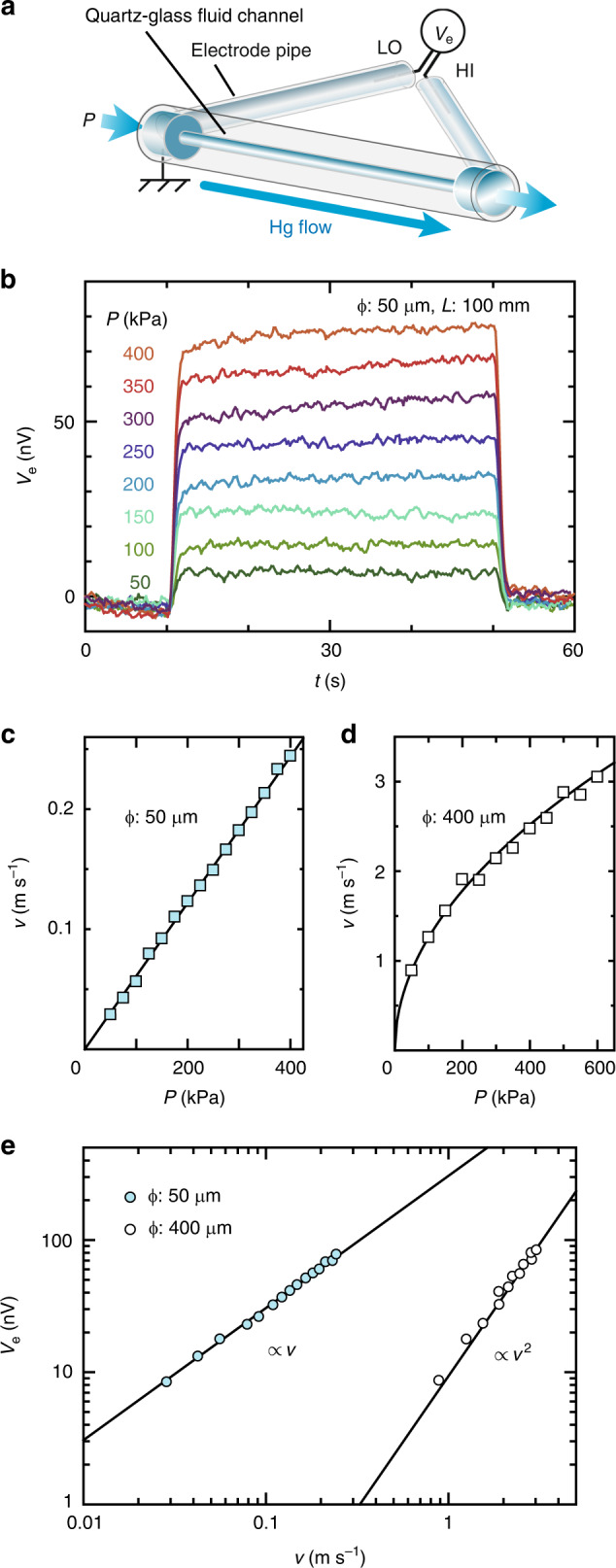
**Measurements of hydrodynamic voltage generation**. **a** A schematic illustration of the fluid channel used in the present study. A flow of liquid Hg in a quartz-glass fluid channel is induced by applying pulsed pressure, *P*. *V*_e_ denotes electric voltage difference between the ends of the channel. HI and LO denote the high and low terminals, respectively. **b** Time evolution of *V*_e_ for various values of *P* in the fluid channel whose inner diameter *ϕ* is 50 μm and the length *L* is 100 mm. **c** *P* dependences of the mean flow velocity *v* for the channel used in (**b**). *v* is estimated by averaging the data obtained from repetitive measurements. The experimental results are well reproduced by a relation for laminar flow states: *v* ∝ *P*. **d** *P* dependence of *v* for the channel of *ϕ* = 400 μm^12^, reproduced well by a relation for turbulent flow states: $v\propto \sqrt{P}$. **e** *v* dependences of *V*_e_ for the channels of *ϕ* = 50 μm (blue filled circles) and *ϕ* = 400 μm (open circles)^12^. *V*_e_ (blue filled circle) is obtained by averaging the data obtained from repetitive measurements. In each measurement, *V*_e_ is estimated by taking a time average. The results obey the SHDG scaling law for laminar flow states: *V*_e_ ∝ *v*, totally different from that for turbulent one: *V*_e_ ∝ *v*^2^.

 Figure 2a shows a schematic illustration of the fluid channel used in the present study. In our study, we generate a flow of a liquid-metal mercury (Hg) in cylindrical quartz-glass channels (length *L* = 100 mm) by applying pulsed pressure, *P*. The inner diameter *ϕ* of the channels is 50, 70, 90, 112, and 126 μm to realize a laminar flow condition, and 200 μm to realize a transition flow condition. Two extra quartz-glass pipes (inner diameter: 1.0 mm, length: 100 mm) filled with Hg are used as electrodes, named electrode pipes shown in Fig. 2a. The electrode pipes are connected to liquid reservoirs near the inlet and the outlet of the fluid channel. The ends of the electrode pipes are placed in close proximity to each other to realize an isothermal condition and are connected to a nanovoltmeter, with which we measured the electric voltage difference *V*_e_. This set-up enables us to avoid thermoelectric disturbance in the measurements because any thermoelectric voltage did not appear in the previous work^12^ when we made a temperature gradient along the channel by applying much greater heat at the end of the channel than that induced by the pulsed pressure. To prevent Hg from charging up electrically, Hg is connected to the ground at the inlet. Furthermore, this set-up enables us to avoid a charging effect, that is contact electrification^22–24^ at the channel wall, because it was experimentally confirmed^12^ that the voltage induced by the Hg flow is almost independent of the electrification property at the contact surface.

### SHDG measurement in laminar flow regime

 Figure 2b shows the time evolution of *V*_e_ measured for various values of *P* in a channel with *ϕ* = 50 μm. The Hg flow in the channel starts at time *t* = 10 s and ends at *t* = 50 s. A clear *V*_e_ signal appears when Hg is flowing. The magnitude of *V*_e_ increases with increasing *P*. Figure 2c shows the mean flow velocity *v* as a function of *P* in the channel. The linear relation between *v* and *P* (*v* ∝ *P*) indicates that the liquid flow in the measurement is in a laminar flow state according to a fluid theory, being consistent with the fact that the Reynolds number of the present flow, Re(=*vϕ*/ν), satisfies the laminar flow condition, 2 < Re < 110, where ν is the kinetic viscosity: ν = 1.2 × 10^−7^ m^2^s^−1^ for Hg^12^. On the other hand, in a turbulent condition, the *P* dependence of *v* exhibits square-root relations ($v\propto \sqrt{P}$) as shown in Fig. 2d. The result indicates that SHDG appears even in laminar flow condition.

In Fig. 2e, we plot the *v* dependence of *V*_e_ in the channels with *ϕ* = 50 μm and *ϕ* = 400 μm. The result for the *ϕ*-50-μm channel clearly illustrates that the *V*_e_ signal is proportional to *v* (*V*_e_ ∝  *v*) in the laminar flow condition. This relation in the laminar flow makes a sharp contrast with the turbulent condition^12^, where *V*_e_ is almost proportional to the square of the mean flow velocity, *V*_e_ ∝ *v*^2^, as shown by a result for the *ϕ*-400-μm channel (Fig. 2e). In fact, such linear dependence of *V*_e_ (*V*_e_ ∝ *v*) is very the feature predicted for SHDG in a laminar flow. The difference of the *v* dependence of *V*_e_ is due to the difference of spatial distribution of  ∇***ω*** between the laminar and turbulent conditions. The gradient of the vorticity in a laminar flow in a cylindrical channel is $\partial {\omega }_{\theta }/\partial r=4v/{{r}_{0}}^{2}$ in a Hagen–Poiseuille flow condition, where *θ* is the azimuthal direction perpendicular to the flow and *r*_0_ ≡ *ϕ*/2. Therefore, in accordance with the SHDG theory^13^, electric voltage in a laminar flow is given by

2 $${V}_{{\rm{e}}}=\frac{8| e| L}{{\sigma }_{0}\hbar }\left({\theta }_{{\rm{SHE}}}{\lambda }^{2}\xi \right)\frac{v}{{{r}_{0}}^{2}},$$

where *θ*_SHE_ is the spin Hall angle. In Eq. (2), *V*_e_ is predicted to be proportional to *v* in a laminar flow, being consistent with the experimental result shown in Fig. 2e.

### SHDG measurements for various values of channel inner diameter

**Fig. 3:**
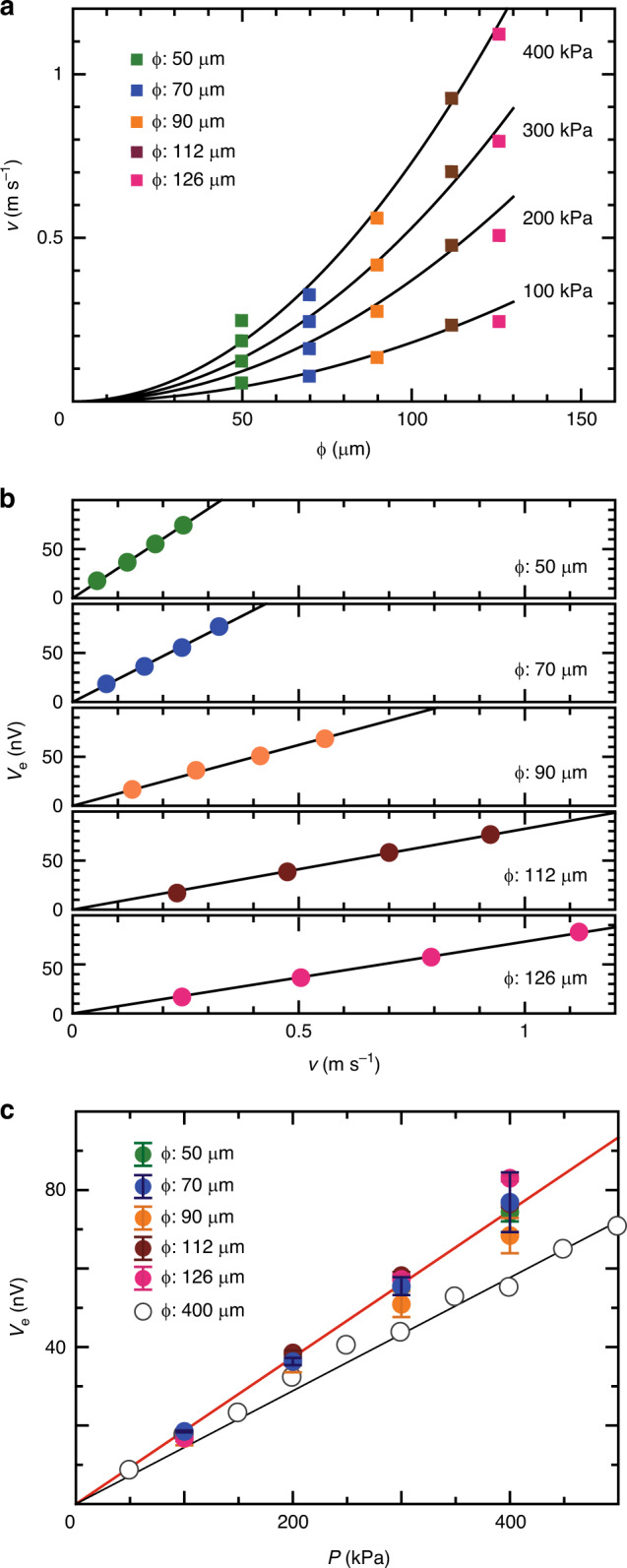
**Voltage measurement in laminar flows for channels**. **a** *ϕ* dependences of *v* for several values of *P*. Dimensional tolerance with respect to *ϕ* is 5 μm for the channels whose *ϕ* are 50, 112 and 126 μm, and 10 μm for the channels whose *ϕ* are 70 and 90 μm, respectively. The *v* data for each *P* is consistent with the relation for a laminar flow: $v\propto {{r}_{0}}^{2}$, indicated by black curves. **b** *v* dependences of *V*_e_ for several values of *ϕ*. **c** *P* dependence of *V*_e_. The open circles are the data of *ϕ*-400-μm channel^12^. The red solid line is the fit line obtained from the equation *V*_e_ = *CP* and the black solid line is an approximate line for the turbulent-flow SHDG. The values of *v* and *V*_e_ in (**a**–**c**) were obtained by averaging the data obtained from 60-times repetitive measurements. Error bars in (**a**) and (**b**) are smaller than the marker sizes and those in (**c**) represent one standard error of the mean in the repetitive measurements.

To further examine the voltage signals, we measured *V*_e_ for various values of *ϕ*. Fig. 3a shows the *ϕ* dependence of *v* for various values of *P*. The solid curves for each pressure shown in Fig. 3a illustrate the relation $v\propto {{r}_{0}}^{2}$, representing the theoretical prediction for laminar flows. Figure 3b shows the *v* dependence of *V*_e_ for channels with different *ϕ* values. The result shows that *V*_e_ increases linearly with *v* but with different slopes for the different *ϕ* values. However, as shown in Fig. 3c, all the different data collapse into a single curve when we plot them in a *V*_e_ – *P* scale. The result indicates that *V*_e_ is proportional to *P* with an identical slope independent of *r*_0_. Since $v\propto {{r}_{0}}^{2}P$ is realized in the laminar (Hagen–Poiseuille) flow condition, as shown in Figs. 2c and 3a, Eq. (2) can be rewritten as *V*_e_ = *CP*, where *C* is a factor independent of *r*_0_, $C=\frac{| e| }{{\sigma }_{0}\hbar \mu }\left({\theta }_{{\rm{SHE}}}{\lambda }^{2}\xi \right)$. Here, *μ* is the Newtonian viscosity. The result is well consistent with the prediction of SHDG in a laminar flow as shown in Eq. (2). By fitting the data in Fig. 3c with the equation of *V*_e_ = *CP*, the parameter *θ*_SHE_*λ*^2^*ξ* is estimated to be 1.9 × 10^−25^ J s m^−1^. Here, we used parameters for Hg^12^, *σ*_0_ = 1.01 × 10^6^ Ω^−1 ^m^−1^ and *μ* = 1.6 × 10^−3^ J s m^−3^. The open circles shown in Fig. 3c are the data for the *ϕ*-400-μm channel, where the turbulent flow is realized. As shown in Fig. 3c, *V*_e_ induced by the SHDG for the turbulent flow is also proportional to *P* but slopes of the lines are different between the laminar and the turbulent flow, resulting from the difference in the velocity distributions.

### SHDG measurement in transition flow regime

**Fig. 4:**
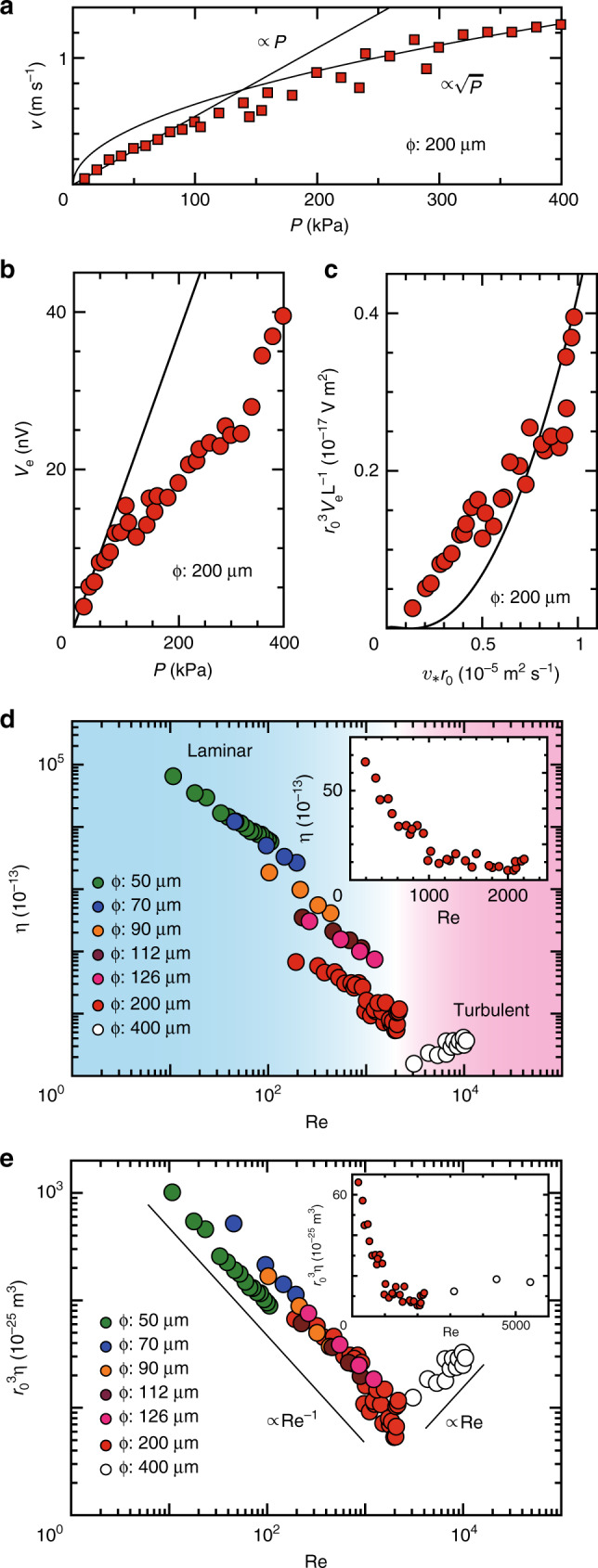
**Voltage measurement in transition flow and conversion efficiency**. **a** *P* dependence of *v* for the channel of *ϕ* = 200 μm. *v* is estimated by averaging the data obtained from repetitive measurements. The experimental results are well reproduced by a relation for laminar flow states in low *P* region (*v* ∝ *P*) and a relation for turbulent flow states in high *P* region ($v\propto \sqrt{P}$). **b** *P* dependence of *V*_e_ for the channel used in (**a**). *V*_e_ is estimated by averaging the data obtained from repetitive measurements. The black solid line represents the theoretical calculation of the laminar-flow SHDG shown in Fig. 3c (red solid line). **c** *v*_∗_*r*_0_ vs. ${{r}_{0}}^{3}{V}_{{\rm{e}}}{L}^{-1}$ plot. *v*_∗_ and *r*_0_ represent the friction-velocity and the inner radius, respectively. The black solid curve represents the theoretical calculation of the turbulent-flow SHDG shown in^12^. **d** Dependence of the energy conversion efficiency *η* on the Reynolds number Re for various values of *ϕ*. *η* estimated from the experimental results of the present study, indicated by filled circles in the laminar and the transition region, are shown together with that in the turbulent region^12^ (open circles). Inset shows Re dependence of *η* for the *ϕ*-200-μm channel with a linear scale. **e** Re vs. ${{r}_{0}}^{3}\eta$ plot for various values of *ϕ*. The black solid lines represent the relations of ${{r}_{0}}^{3}\eta \propto {\rm{Re}}^{-1}$ and ${{r}_{0}}^{3}\eta \propto {\rm{Re}}$. Inset shows Re vs. ${{r}_{0}}^{3}\eta$ plot for the *ϕ*-200-μm and the *ϕ*-400-μm channels with a linear scale.

We also carried out measurements on the SHDG in a transition flow regime by using the channel with *ϕ* = 200 μm and *L* = 100 mm. In the measurement, Re was modulated between 200 and 2210 only by changing *P*. As shown in Fig. 4a, the relation between *P* and *v* changes from the linear relation to the square-root one with increasing *P*, indicating that the Hg flow changed from the laminar flow regime to the turbulent one in the measurement. The intermediate region of *P* = 150~300 kPa is the transition region from the laminar flow to the turbulent flow, in which the flow seems to be unstable. In Fig. 4b, we show the *P* dependence of *V*_e_. In low *P* region, *V*_e_ is in good agreement with the black solid line, which is the theoretical calculation of the laminar-flow SHDG using *θ*_SHE_*λ*^2^*ξ* = 1.9 × 10^−25^ J s m^−1^ estimated from the fit to the data in Fig. 3c. In Fig. 4c, we show the *v*_∗_*r*_0_ vs. ${{r}_{0}}^{3}{V}_{{\rm{e}}}{L}^{-1}$ plot so as to discuss the turbulent flow, where *v*_∗_ is the friction-velocity^21^. ${{r}_{0}}^{3}{V}_{{\rm{e}}}{L}^{-1}$ in the high *v*_∗_*r*_0_ region, namely the high velocity region, is in good agreement with the black solid curve, which is the theoretical calculation of the turbulent-flow SHDG using^12^ *θ*_SHE_*λ*^2^*ξ* = 5.9 × 10^−25^ J s m^−1^. The above results indicate that the low and high Re region are consistent with the SHDG theory of the laminar and turbulent flow, respectively, and that the SHDG voltage continuously changes from the laminar behavior to the turbulent one with changing Re across the transition flow regime.

### Estimation of energy conversion efficiency

Finally, we estimate the energy conversion efficiency of the present SHDG. In the estimation, the energy injection per unit time *W*_in_ is assumed to be same as the kinematic energy gain per unit time calculated by using *v*: ${W}_{{\rm{i}}\mathrm{n}}=\rho \pi {{r}_{0}}^{2}{v}^{3}/2$, where *ρ* is the mass density. Energy generation per unit time via SHDG, *W*_out_, is estimated as the electric power: ${W}_{{\rm{out}}}=\pi {{r}_{0}}^{2}{\sigma }_{0}{{V}_{\rm{e}}}^{2}/L$, and thus the energy efficiency *η* is estimated to be *W*_out_/*W*_in_. Figure 4d shows the Re dependence of *η*, indicating significant improvement of the efficiency in the laminar state; the laminar-flow SHDG is much more efficient than the turbulent-flow SHDG. The maximum magnitude of *η* in the laminar flow in our set-up is 10^5^ times greater than that in the turbulent flow: giant SHDG. A flow with smaller Reynolds number, that is, a slower flow in a smaller channel, should be more efficient in the SHDG energy conversion. If we prepare a sample with *L* ~ 1 mm, *r*_0_ ~ 1 μm and Re ~ 10^−6^, which can be realized by using microfluidic devices, we can realize efficiency about 10%. It is to be noted that the data in Fig. 4d seems to jump by changing Re. This is because *η* depends on not only Re but also *r*_0_ as follows: $\eta \propto L\,{{r}_{0}}^{-3}\mathrm{R{e}}^{-1}$ in the laminar flow regime and $\eta \sim L\,{{r}_{0}}^{-3}\mathrm{Re}$ in the turbulent one.

### Examination of continuity of SHDG phenomenon

The inset in Fig. 4d shows Re dependence of *η* for the *ϕ*-200-μm channel with a linear scale. In the low Re region (Re < 1000), *η* decreases with increasing Re. This behavior is consistent with the results of the laminar flow. In the high Re region (Re > 2000), however, *η* increases with increasing Re. This behavior is also consistent with the results of the turbulent flow. In the intermediate region (1000 < Re < 2000), *η* is almost independent of Re. The result indicates that *η* is continuous across the region between the laminar and turbulent flow. To further examine the continuity of the SHDG phenomenon, we plot Re dependences of ${{r}_{0}}^{3}\eta$ for various values of *ϕ* in Fig. 4e. In the Re – ${{r}_{0}}^{3}\eta$ scale, we could deal with the SHDG phenomenon without considering the direct dependence on *r*_0_ because of the theoretical prediction of *η* described above. The result shows that ${{r}_{0}}^{3}\eta$ is proportional to Re^−1^ in the laminar flow regime, while it is proportional to Re in the turbulent one, being consistent with the SHDG theory. In the inset in Fig. 4e, we replot Re dependences of ${{r}_{0}}^{3}\eta$ for the *ϕ*-200-μm and *ϕ*-400-μm channels with a linear scale. The result also indicates the continuity of the SHDG phenomenon in the laminar-to-turbulent crossover regime because there is no measurable data jump and the values of ${{r}_{0}}^{3}\eta$ seem to change smoothly from the laminar behavior to the turbulent one. Therefore, all the results suggest that the SHDG phenomenon changes continuously from the laminar to the turbulent one across the laminar-to-turbulent crossover regime.

## Discussion

Our finding highlights the energy conversion efficiency of the SHDG, revealing that the SHDG in the laminar flow is much more efficient than that in the turbulent flow. From application points of view, the SHDG has also unique advantages: the voltage generation dependent on the flow rate as shown in Eq. (2) and the undisturbance to the flow because of the absence of accessories in the channel to measure the flow rate. These advantages may enable us to make a miniaturized flow sensor for a liquid metal, which could be used, for example, as an active cooling system in a fast-breeder reactor, a semiconductor device and so on.

## Methods

### Experimental set-up

In the present study, we measured the electric voltage difference *V*_e_ between the ends of the cylindrical quartz-glass channel (Nakahara Opto-Electronics Laboratories, Inc.) filled with the liquid-metal mercury (Hg, 99.5%; Wako Pure Chemical Industries, Ltd.) by using a nanovoltmeter (2182A; Keithley Instruments, Inc.) while measuring the amount of Hg discharged from the channel simultaneously to estimate the mean flow velocity *v* by using an electronic balance (HR-250AZ; A&D, Co., Ltd.). To generate a flow of Hg, we applied the pulsed pressure *P* to the liquid reservoir connected to the channel inlet by using a pressure controller (PACE5000; GE Sensing & Inspection Technologies, Co., Ltd.). All the measurements were performed at room temperature.

## Supplementary information

Supplementary Information

## Data Availability

All relevant data are available from the corresponding author upon reasonable request.
