# Supplementary material for: Giant spin hydrodynamic generation in laminar flow
Source: Nat Commun. 2020 Jun 15;11:3009. doi: 10.1038/s41467-020-16753-0 (PMC7295809; doi:10.1038/s41467-020-16753-0)
Supplement: Supplementary file 1 — Supplementary Information [file 41467_2020_16753_MOESM1_ESM.pdf]

Reviewers' comments:

Reviewer #1 (Remarks to the Author):

The manuscript “Giant spin hydrodynamic generation in laminar flow” presents an experimental study of flow-induced electric field generation in mercury-filled quartz capillaries. The main experimental observation is a linear relation between differential pressure applied to the capillary and electric voltage induced across the capillary. The novel aspect of this work is that laminar mercury flow is studied while the previous work (Ref. [1]) studied the turbulent flow regime. Mercury flow through capillaries of different diameters (some operating in laminar regime and one operating in turbulent regime) was experimentally studied.

The authors claim that the observed voltage arises from a pure spin current created by spin-rotation coupling in flowing mercury. This spin current is then converted into an electric voltage via the inverse spin Hall effect. More specifically, the authors claim that efficiency of converting the fluid mass flow into inverse spin Hall voltage is higher in the laminar flow regime compared to the turbulent flow regime. This is the main result of the paper.

While I find that the claims made in the paper to be plausible, the experimental evidence to support these claims is not quite convincing, and thus additional measurements are necessary to either prove or disprove these claims.

My main concern is that the authors did not study the crossover between laminar and turbulent flow in the same capillary. The authors studied either capillaries of small diameter (< 0.13 mm) where only laminar flow is achieved or a capillary of large diameter (0.4 mm) where only turbulent flow is studied. I think it is imperative to measure flow-induced voltage in capillaries of intermediate diameters where a crossover between laminar and turbulent flow can be observed with increasing pressure and flow rate. If the author’s claim of enhanced efficiency in the laminar flow is true, the induced voltage should switch from liner behaviour as a function of flow rate to sub-linear behaviour upon transition from laminar to turbulent flow in such capillaries of intermediate diameters. In my opinion, this measurement is necessary to validate the author’s main claim.

I also have several minor comments and questions:

- Is there a potential practical application for spin hydrodynamic generation? If yes, it should be discussed in the paper.

- I recommend to add data for the 0.4 mm diameter channel exhibiting turbulent flow to Fig. 3c. Do the data for this wider channel collapse onto the same curve as the data for the narrower channels? A corresponding discussion should be added to the paper.

- The statement “In micro or nano scale devices, a fluid flow inevitably becomes laminar.” is too strong. I agree this is usually true for nanoscale devices but not always correct for micrometer-scale devices.

- In Fig. 4, what is the theoretical prediction for the dependence of efficiency on the Reynolds number? Adding a theoretical curve to this plot would help the reader understand the degree of agreement between theory and experiment.

- It is somewhat disconcerting that all evidence of spin-induced nature of the observed phenomenon is indirect. Can the authors discuss possible direct measurements of spin current origin of the induced

voltage in the discussion part of the paper? For example, the first convincing measurement of spin Hall effect was direct magneto-optical observation of spin accumulation driven by spin Hall effect (Y. K. Kato et al., Science, 306, 1910 (2004)). What would an equivalent “smoking gun” measurement for the spin hydrodynamic generation? I am not asking the authors to make such a measurement, but proposing such a measurement would stimulate further progress in this field.

- Given the lack of direct evidence that the observed voltage is spin-related, a discussion on why other origins of the observed voltage can be eliminated is due. For example, can pressure gradients present at the ends of the capillary induce electric dipole layers and produce small voltages similar to those seen in the experiment? Can pressure-induced voltages in piezoelectric quartz (the channel wall material) be eliminated as sources of the observed voltage etc?

Reviewer #2 (Remarks to the Author):

The manuscript “Giant spin hydrodynamic generation in laminar flow”, by undisclosed authors, seems to contain interesting results on a matter that is both timely and relevant. Before a final decisions is made about the relevance and potential impact of the present piece of work, I ask the authors to consider the following questions, remarks, and suggestions, to improve the quality of their presentation.

1. I am not sure I understand why the close proximity of the two electrode pipes alone may ensure isothermal conditions. May the authors expand their discussion on this crucial issue? If a temperature gradient appears along the flowing pipe, how fast would it relax along the electrode pipes? Is this a sensible way to remove the effect of a thermal gradient in the flowing pipe from the measured electric potential difference?

2. A value and a reference for the kinematic viscosity of Hg are desirable. Similarly, a reference is laking for the value adopted for electrical conductivity of Hg. The symbol \sigma_0 should be explained soon after Eq. (2).

3. What is the size of the error bars on the velocity measurements in Fig. 3?

4. There seems not to be an easy way to connect the low-Re measurements in Fig. 4 with the previous high-Re measurements of the study reported in Ref. [1]. The change across the narrow white-shaded region seems rather abrupt, and the trends on the left and on the right of this crossover region seem to be opposite. Why did the authors avoid to explore the crossover to the turbulent regime within their experimental setting? Is there any simple reason to understand why the slope of the measured conversion efficiency is rather constant, but the data jump when changing the flowing channel diameter?

5. The authors cover two decades of Re, from 10 to 1000 in Fig. 4. How reasonable is it to extrapolate the conversion efficiency to Re=10^{-6}? Is not the final statement really too daring, as it requires an extrapolation over 7 decades?

There are also minor points.

a. I am not sure the sentence “a liquid metal flows in a fluid channel”, on page 3, is meaningful. Did the authors mean “cylindrical channel”?

b. The third panel of Fig. 2 is confusing. While in the text a clear distinction between the symbols V and v exists, in the figure they look the same and are only distinguishable by their size in the axis labels. In the figure panel, however, the v for velocity is the same size as the V for electric voltage difference appearing in the vertical axis label, making the figure hardly readable.

Once the authors have complied with the various issues listed above, I suggest that their manuscript may be reconsidered for publication in Nature Communications.

Reviewer #3 (Remarks to the Author):

This manuscript reports on electrical voltage generation due to a flow of Hg liquid through a micrometer-scale cylindrical channel. The flow gradient inside the channel gives rise to a spin current flow in the radial direction due to spin-rotation coupling, which is ultimately converted into an electrical voltage along the channel by the inverse spin Hall effect. The authors find a substantial enhancement in such a mechanism when the flow is laminar instead of turbulent, owing to the smaller inner diameter of the channel, and call it 'giant' spin hydrodynamic generation (SHDG). The results are neat, the analysis and interpretation of the data are sound and clear.

The SHDG is recently discovered by Prof. Saitoh and colleagues, nicely demonstrating the coupling between mechanical angular momentum and spin angular momentum in a liquid conductor. A few theoretical and experimental studies have followed up this pioneering work, extending the knowledge on this phenomenon. However, the general interest remained limited partly because the practical utility of such a mechanism is not yet apparent (generated signals around ~nV, efficiencies of the order of 10^-13) but also the experiments are restricted to a few systems due to the requirement of liquid conductors at room temperature.

I find this study as a natural extension of the original work without giving significanlty novel physical insight. The experimental setup, the material considerations are the same, except that the tube dimensions are downsized by about one order of magnitude (from 400 to 50 um), which has resulted in similar signal amplitudes (~nV) but in increased efficiency of the overall process (from 10^-13 to ~10^-8) once properly normalized by the pressure flow velocity etc. However, these numbers are still way too small to attract interest from an application viewpoint and are only measurable with dedicated electronics.

Based on the above considerations, I do neither see how this work may have a significant impact on the scientists working in this field nor how a practical application can be envisaged based on these results. Therefore, I do not recommend this manuscript for publication in Nature Communications.

Reviewer #4 (Remarks to the Author):

The present manuscript deals with the spin hydrodynamic generation (SHDG) based on the coupling between mechanical rotation in a fluid and its electron spins. The experimental results show electric voltage generation due to the flux of electron-spin’s angular momentum. The so-called spin hydrodynamic generation is observed in the laminar flow regime that is relevant on a micro- or nano-scale. Furthermore, the presented-data report that the energy conversion efficiency in a laminar flow state is about five orders of magnitude greater than that in the turbulent one.

The topic deals with the emerging field of fluid spintronics. The spin hydrodynamic generation effect

has been already shown in Ref Nat. Phys. 12, 52-56 (2016), Phys. Rev.B 96, 020401(R) (2017). The authors in this work try to extend experimentally the existing published work in the laminar flow regime.

The manuscript is well written.

The main question is whether the measured voltage is only due to the spin current or it can also be attributed to charging effects. The authors should explain more in details why charging effects are not relevant (statement at the end of page 10). The independency of the Voltage signal on P is a natural consequence of the laminar flow state and the Hagen-Poiseuille law, as it is seen from Figure 3. Following this argument, do the authors mean that we should not expect charging effects by any laminar flow?

I propose that the authors should clarify in general why parasitic effects are not present. Is there side walls voltage?

The meaning of the viscosity correction ξ in Equation 1 should be explained. Where does it refer to? The viscosity can cause a diffusion of vorticity away from the vortex cores and this can in principal influence the functionality of a SHDG.

Could the establishment of electric field, E(SHD), lead to parasitic effects? (for example, the occurrence of a Hall effect in their device)

Have the authors measured the transition from turbulent to laminar flow regime?

In spintronics values like θ(SHE), λ are of unique importance. How can they be envisaged in the case of liquid Hg in Equation 2? What is their order of magnitude?

The conversion efficiency shown in Figure 4 is very small. Which factor establishes the maximum theoretical efficiency of a such spin hydrodynamic engine? Although tube lengths of the mm size can be fabricated, are Reynold's numbers of the order of 10-6 realistic? How can this efficiency be compared to the power output of a magneto-hydrodynamic generator?

Reviewer #1:

In the following, we present our point-by-point responses to the reviewer's comments, in which the original comments are reproduced in italics.

The manuscript "Giant spin hydrodynamic generation in laminar flow" presents an experimental study of flow-induced electric field generation in mercury-filled quartz capillaries. The main experimental observation is a linear relation between differential pressure applied to the capillary and electric voltage induced across the capillary. The novel aspect of this work is that laminar mercury flow is studied while the previous work (Ref. [1]) studied the turbulent flow regime. Mercury flow through capillaries of different diameters (some operating in laminar regime and one operating in turbulent regime) was experimentally studied.

The authors claim that the observed voltage arises from a pure spin current created by spin-rotation coupling in flowing mercury. This spin current is then converted into an electric voltage via the inverse spin Hall effect. More specifically, the authors claim that efficiency of converting the fluid mass flow into inverse spin Hall voltage is higher in the laminar flow regime compared to the turbulent flow regime. This is the main result of the paper.

While I find that the claims made in the paper to be plausible, the experimental evidence to support these claims is not quite convincing, and thus additional measurements are necessary to either prove or disprove these claims.

Q1.
My main concern is that the authors did not study the crossover between laminar and turbulent flow in the same capillary. The authors studied either capillaries of small diameter (< 0.13 mm) where only laminar flow is achieved or a capillary of large diameter (0.4 mm) where only turbulent flow is studied. I think it is imperative to measure flow-induced voltage in capillaries of intermediate diameters where a crossover between laminar and turbulent flow can be observed with increasing pressure and flow rate. If the author's claim of enhanced efficiency in the laminar flow is true, the induced voltage should switch from liner behavior as a function of flow rate to sub-linear behavior upon transition from laminar to turbulent flow in such capillaries of intermediate diameters. In my opinion, this measurement is

necessary to validate the author's main claim.

A1.

Following the referee's comments, we carried out the additional experiments using a capillary of the diameter ϕ = 200 μm. realizing a crossover between the laminar and turbulent flow. In this measurement, *Re* can be varied from 200 to 2,210 with the pressure *P*.

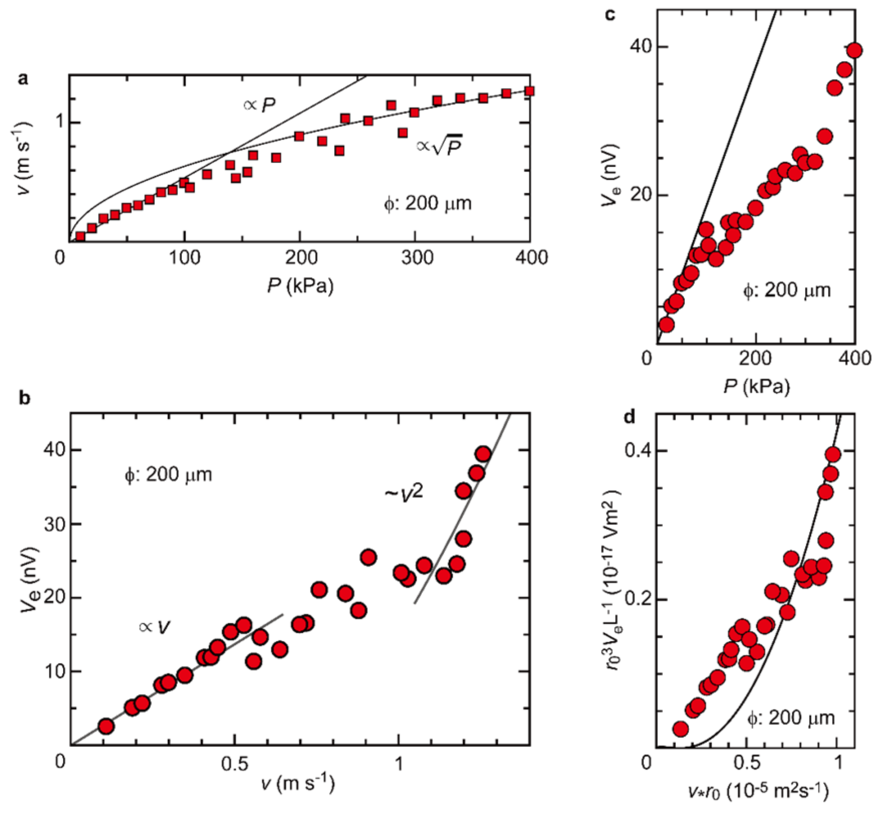

Fig. R1.1

The results are shown in Fig. R1.1. As shown in Fig. R1.1a, the *P* dependence of the mean velocity *v* changes from $\propto P$ in low *P* region to $\propto\sqrt{P}$ in high *P* region, indicating that the laminar flow in the low *P* region is converted into the turbulent flow in the high *P* region. In Fig. R1.1b, we show the *v* dependence of the voltage V_e. In the plot, V_e shows crossover from $\propto v$ in the low *v* region to $\propto v^2$ in the high *v* region. The intermediate region of v=0.5~1 ms^{-1} is the transition region from the laminal flow to the turbulent flow. This intermediate region coincides with the region of P=150~300 kPa in Fig. R1.1a.

In Fig. R1.1 c, we show the *P* dependence of V_e. In the low *P* region, V_e is in good agreement with the black solid line, which is the theoretical calculation of the laminar SHDG using $\theta_{\mathrm{SHE}}\lambda^2\xi = 1.9 \times 10^{-25}\,\mathrm{J\,s\,m^{-1}}$ estimated from the fit to the data in Fig. 3c in the main text.

In Fig. R1.1d, we show the $v_* r_0$ vs. $r_0^3 V_e L^{-1}$ plot so as to discuss the turbulent flow, where r_0 and L are the radius and the length of the capillary, respectively. v_* is the friction velocity (refer to p53 in Nat. Phys. **12** 52-56 (2016)). $r_0^3 V_e L^{-1}$ in the high $v_* r_0$ region, namely the high velocity region, is in good agreement with the black solid curve, which is the theoretical calculation of the turbulent SHDG using $\theta_{\mathrm{SHE}}\lambda^2\xi = 5.9 \times 10^{-25}\,\mathrm{J\,s\,m^{-1}}$ estimated from the fit to the data in Fig. 2f in the Nat. Phys. **12** 52-56 (2016).

Above results indicate that the low and high *Re* region are consistent with the SHDG theory of the laminar and turbulent flow, respectively.

We added these results and the corresponding sentences to the main text.

I also have several minor comments and questions:

Q2.

- Is there a potential practical application for spin hydrodynamic generation? If yes, it should be discussed in the paper.

A2.

From application points of view, the SHDG has unique advantages as follows. The SHDG in the laminar flow is much more e□cient than that in the turbulent flow. The voltage V_e induced by the SHDG depends on the flow rate as shown in eq. (2) in the main text. Furthermore, SHDG does not disturb the flow because of the absence of accessories in the channel to measure the flow rate. These advantages of the SHDG may enable us to make a miniaturized flow sensor for a cooling system using liquid metal such as a fast-breeder reactor.

We added the corresponding paragraph to the main text.

Q3.

- I recommend to add data for the 0.4 mm diameter channel exhibiting turbulent flow to Fig. 3c. Do the data for this wider channel collapse onto the same curve as the data for the narrower channels? A corresponding discussion should be added to the paper.

A3.

Following the referee's recommendation, we added the data for ϕ = 400 µm, where the turbulent flow is realized, to Fig. 3c in the main text. As shown in Fig. R1.2, V_e induced by SHDG for the turbulent flow also seems to be proportional to P. But the slopes of the lines are different between the laminar and turbulent flow. The reason is that the distributions of the flowing velocity are different between the laminar and turbulent flow. More specifically, an approximate equation of the slope on the turbulent flow regime includes phenomenological coefficients on the turbulent flow such as the von Karman's constant, the Darcy friction factor and so on.

We also add the corresponding discussion to the main text.

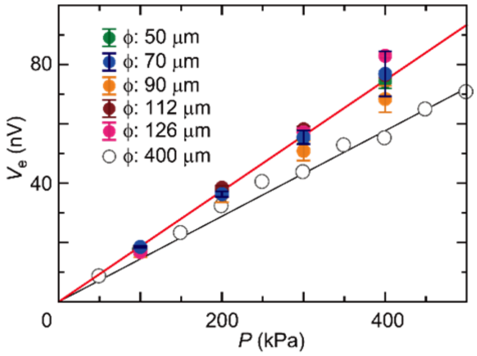

Fig. R1.2

Q4.

- The statement "In micro or nano scale devices, a fluid flow inevitably becomes laminar." is too strong. I agree this is usually true for nanoscale devices but not always correct for micrometer-scale devices.

A.4

Following the referee's comments, we changes the corresponding sentence to "a fluid flow usually becomes laminar".

Q.5

- In Fig. 4, what is the theoretical prediction for the dependence of efficiency on the Reynolds number? Adding a theoretical curve to this plot would help the reader understand the degree of agreement between theory and experiment.

A.5

The efficiency η is defined as,

$$\eta \equiv \frac{W_{\mathrm{out}}}{W_{\mathrm{in}}} = \frac{2\sigma_0 {V_e}^2}{\rho v^3 L},$$

in the main text. By substituting V_e into eq. (2), η can be written as

$$\eta = \frac{(16e)^2 L}{\mu \sigma_0 \hbar^2} {K_0}^2 \frac{1}{{r_0}^3 \mathcal{R}e},$$

where, $K_0 \equiv \theta_{\mathrm{SHE}} \lambda^2 \xi$. As shown in the equation, η depends not only on *Re* but also on r_0. Therefore, we need plural curves depending on various r_0 so as to display the theoretical curves on Fig. 4 in the main text. We are afraid that we do not display the theoretical curves on Fig. 4 so as not to complicate the appearance of Fig.4 in the main text.

Q.6

- It is somewhat disconcerting that all evidence of spin-induced nature of the observed phenomenon is indirect. Can the authors discuss possible direct measurements of spin current origin of the induced voltage in the discussion part of the paper? For example, the first convincing measurement of spin Hall effect was direct magneto-optical observation of spin accumulation driven by spin Hall effect (Y. K. Kato et al., Science, 306, 1910 (2004)). What would an equivalent "smoking gun" measurement for the spin hydrodynamic generation? I am not asking the authors to make such a measurement, but proposing such a measurement would stimulate further progress in this field.

A.6

We have been also seeking techniques to directly measure the spin accumulation at the surface of the flowing liquid metal. We need to expose the surface of liquid flow to air so as to measure the spin accumulation using a conventional method established in a solid sample. We have not come up with the experimental setup to realize above condition. Therefore, we have not been able to do such an experiment yet.

Q.7

- Given the lack of direct evidence that the observed voltage is spin-related, a discussion on why other origins of the observed voltage can be eliminated is due. For example, can pressure gradients present at the ends of the capillary induce electric

dipole layers and produce small voltages similar to those seen in the experiment? Can pressure-induced voltages in piezoelectric quartz (the channel wall material) be eliminated as sources of the observed voltage etc?

A.7

We are sorry for confusing you. To be exact, we used quartz-“glass” capillaries, not crystalline quartz capillaries. Therefore, any voltage induced by the piezo effect are not generated. We corrected the corresponding sentence in the main text.

Reviewer #2:

In the following, we present our point-by-point responses to the reviewer's comments, in which the original comments are reproduced in italics.

The manuscript "Giant spin hydrodynamic generation in laminar flow", by undisclosed authors, seems to contain interesting results on a matter that is both timely and relevant. Before a final decisions is made about the relevance and potential impact of the present piece of work, I ask the authors to consider the following questions, remarks, and suggestions, to improve the quality of their presentation.

Q.1

I am not sure I understand why the close proximity of the two electrode pipes alone may ensure isothermal conditions. May the authors expand their discussion on this crucial issue? If a temperature gradient appears along the flowing pipe, how fast would it relax along the electrode pipes? Is this a sensible way to remove the effect of a thermal gradient in the flowing pipe from the measured electric potential difference?

A.1

The experimental setup used in this article is the same as the setup in the previous work (Nat. Phys. **12** 52-56 (2016)). As shown in Fig. 4b in Nat. Phys. **12** 52-56 (2016), we made a temperature gradient on the capillary with much greater heat at the end of capillary than that induced by the pulsed pressure so as to investigate the thermoelectric effect. The result is that any voltage does not appear in this experiment, which indicates that isothermal condition between electrodes is established well in this setup.

We added the corresponding sentence to the main text.

Q.2

A value and a reference for the kinematic viscosity of Hg are desirable. Similarly, a reference is lacking for the value adopted for electrical conductivity of Hg. The symbol ¥sigma_0 should be explained soon after Eq. (2).

A.2

Thank you for your kind indications. Following the comment, we provided the

value of the viscosity and conductivity of Hg, and these references. We are afraid that σ_0 is already explained just after eq. (1) in the main text.

Q3.

What is the size of the error bars on the velocity measurements in Fig. 3?

A.3

The definition of the error bars on the velocity measurements in Fig. 3a is the 1σ on 60 times measurements at each diameter of the capillaries and the pressures. Since the error bars on the velocity measurements in Fig. 3a are smaller than the marker size, we omitted the error bars.

We added the corresponding sentence to the caption of Fig. 3 in the main text.

Q.4

There seems not to be an easy way to connect the low-Re measurements in Fig. 4 with the previous high-Re measurements of the study reported in Ref. [1]. The change across the narrow white-shaded region seems rather abrupt, and the trends on the left and on the right of this crossover region seem to be opposite. Why did the authors avoid to explore the crossover to the turbulent regime within their experimental setting? Is there any simple reason to understand why the slope of the measured conversion efficiency is rather constant, but the data jump when changing the flowing channel diameter?

A.4

Following the referee's comments, we carried out the additional experiments using a capillary of the diameter ϕ = 200 μm realizing a crossover between the laminar and turbulent flow. In this measurement, *Re* can be varied from 200 to 2,210 with the pressure *P*. We added the results to Fig. 4d in the main text as shown in Fig. R2.2. The inset in Fig. R2.2 shows *Re* dependence of η for the ϕ-200-μm capillary with a linear scale. In the low *Re* region (*Re*<1,000), η decreases with increasing *Re*. This behavior is consistent with the results of the laminar flow. In the high *Re* region (*Re*>2,000), however, η increases with increasing *Re*. This behavior is also consistent with the results of the turbulent flow. In the intermediate region (1,000<*Re*<2,000), η is almost independent of *Re* and continuous across the region between the laminar and turbulent flow.

The reason for the data jumping by changing the channel diameter is that η

depends on the radius of the cannel r_0. The η for the laminar flow regime is in the following:

$$\eta = \frac{(16e)^2 L}{\mu \sigma_0 \hbar^2} {K_0}^2 \frac{1}{{r_0}^3 \mathcal{R}e},$$

where, $K_0 \equiv \theta_{\mathrm{SHE}} \lambda^2 \xi$. Therefore, the theoretical curves on *Re* dependence of η are different in r_0 from one another.

We added the data on the crossover region to Fig.4 and revised the figures. We also added the corresponding paragraph and sentences to the main text.

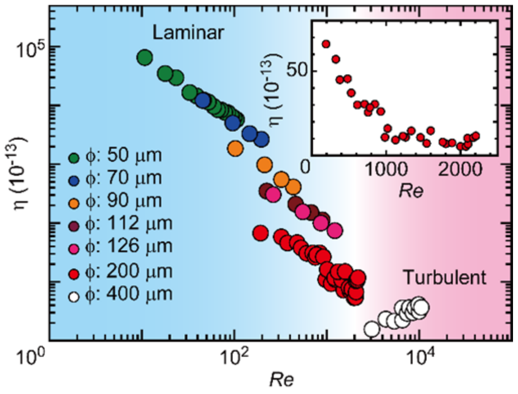

Fig. R2.2

Q.5

The authors cover two decades of Re, from 10 to 1000 in Fig. 4. How reasonable is it to extrapolate the conversion efficiency to Re=10^{-6}? Is not the final statement really too daring, as it requires an extrapolation over 7 decades?

A.5

Re=10^{-6} can be realized by using microfluidic devices. We have made a microfluidic device with the hydraulic diameter of ~5 μm and the length of ~1 mm, and have flowed Hg with a velocity of ~0.1 μm/s for the purpose of another experiment. *Re* is estimated to be 10^{-6} order using this device. However, unfortunately, we have not explored SHDG using this device yet, because we have not been able to overcome the difficulty of attaching the electrodes to this small device retaining the isothermal condition.

The flow of Hg should follow the Navier-Stokes equation even though the size of the channel is in micro-meter order. On the other hand, from the point of view of the spin current, SHDG should follow the spin-diffusion equation expressed in eq.

(1) in Nat. Phys. **12** 52-56 (2016), when the diameter of the channel is larger than the spin-diffusion length (10~100 nm for Hg, probably). Thus, SHDG is thought to be realized using the microfluidic device with $Re=10^{-6}$.

There are also minor points.

Q.6

I am not sure the sentence "a liquid metal flows in a fluid channel", on page 3, is meaningful. Did the authors mean "cylindrical channel"?

A.6

Thank you for your indication. We revised the corresponding statement.

Q.7

The third panel of Fig. 2 is confusing. While in the text a clear distinction between the symbols V and v exists, in the figure they look the same and are only distinguishable by their size in the axis labels. In the figure panel, however, the v for velocity is the same size as the V for electric voltage difference appearing in the vertical axis label, making the figure hardly readable.

A.7

Thank you for your indication. We converted V into V_e in the manuscript.

Once the authors have complied with the various issues listed above, I suggest that their manuscript may be reconsidered for publication in Nature Communications.

Reviewer #3:

In the following, we present our response to the reviewer's comment, in which the original comments are reproduced in italics.

This manuscript reports on electrical voltage generation due to a flow of Hg liquid through a micrometer-scale cylindrical channel. The flow gradient inside the channel gives rise to a spin current flow in the radial direction due to spin-rotation coupling, which is ultimately converted into an electrical voltage along the channel by the inverse spin Hall effect. The authors find a substantial enhancement in such a mechanism when the flow is laminar instead of turbulent, owing to the smaller inner diameter of the channel, and call it 'giant' spin hydrodynamic generation (SHDG). The results are neat, the analysis and interpretation of the data are sound and clear.

The SHDG is recently discovered by Prof. Saitoh and colleagues, nicely demonstrating the coupling between mechanical angular momentum and spin angular momentum in a liquid conductor. A few theoretical and experimental studies have followed up this pioneering work, extending the knowledge on this phenomenon. However, the general interest remained limited partly because the practical utility of such a mechanism is not yet apparent (generated signals around ~nV, efficiencies of the order of 10^-13) but also the experiments are restricted to a few systems due to the requirement of liquid conductors at room temperature.

Comment

I find this study as a natural extension of the original work without giving significantly novel physical insight. The experimental setup, the material considerations are the same, except that the tube dimensions are downsized by about one order of magnitude (from 400 to 50 um), which has resulted in similar signal amplitudes (~nV) but in increased efficiency of the overall process (from 10^-13 to ~10^-8) once properly normalized by the pressure flow velocity etc. However, these numbers are still way too small to attract interest from an application viewpoint and are only measurable with dedicated electronics.

Answer

The SHDG in the laminar flow is not a natural extension of the turbulent flow because a laminar flow and a turbulent flow have completely different physics.

In particular, turbulent flow is chaotic, and thus, treated statistically while laminar flow is deterministic so that the Navier-Stokes equation can be solved analytically. As a matter of fact, we demonstrated the different scaling across from the laminar to turbulent flow with the theoretical prediction of the SHDG. This is a nontrivial behavior. Although we agree with the importance of the magnitude of SHDG signal, it is out of scope of this work.

As for the application, the biggest advantage of the SHDG is that the flow speed can be measured without disturbing the flow in itself unlike a conventional flow meter using a propeller or a float. The SHDG may be applicable to a miniaturized flow sensor for a liquid metal, which could be used, for example, as an active cooling system in a fast-breeder reactor, a semiconductor device and so on.

Based on the above considerations, I do neither see how this work may have a significant impact on the scientists working in this field nor how a practical application can be envisaged based on these results. Therefore, I do not recommend this manuscript for publication in Nature Communications.

Reviewer #4:

In the following, we present our point-by-point responses to the reviewer's comments, in which the original comments are reproduced in italics.

The present manuscript deals with the spin hydrodynamic generation (SHDG) based on the coupling between mechanical rotation in a fluid and its electron spins. The experimental results show electric voltage generation due to the flux of electron-spin's angular momentum. The so-called spin hydrodynamic generation is observed in the laminar flow regime that is relevant on a micro- or nano-scale. Furthermore, the presented-data report that the energy conversion efficiency in a laminar flow state is about five orders of magnitude greater than that in the turbulent one.

The topic deals with the emerging field of fluid spintronics. The spin hydrodynamic generation effect has been already shown in Ref Nat. Phys. 12, 52-56 (2016), Phys. Rev.B 96, 020401(R) (2017). The authors in this work try to extend experimentally the existing published work in the laminar flow regime.

The manuscript is well written.

Q.1

The main question is whether the measured voltage is only due to the spin current or it can also be attributed to charging effects. The authors should explain more in details why charging effects are not relevant (statement at the end of page 10). The independency of the Voltage signal on P is a natural consequence of the laminar flow state and the Hagen-Poiseuille law, as it is seen from Figure 3. Following this argument, do the authors mean that we should not expect charging effects by any laminar flow?

I propose that the authors should clarify in general why parasitic effects are not present. Is there side walls voltage?

A.1

Following the reviewer's comment, we added the sentence about our previous work, in which we excluded the voltage arising from other phenomena in our setup.

At the interface between Hg and capillary, the contact electrification should arise. When the Hg flows, the charging of Hg at the interface due to the contact electrification is dragged by the flow such as the slip at the interface and generate the charge current. The sign of this charge current is determined by the difference of work function between Hg and the channel. In previous study in Nat. Phys. **12**, 52-56 (2016), we had carried out the experiment to examine this charging effect (Fig. 3d in Nat. Phys. **12**, 52-56 (2016)). The inside wall of the capillary was coated with the resin to change the electrification property of Hg. The result is that the sign or magnitude of the voltage induced by the Hg flow is almost unchanged, which indicates that the charging effect at the interface is eliminated.

We had also examined the thermoelectric effect in the previous work. The experimental setup used in this article is the same as the setup in Nat. Phys. **12** 52-56 (2016). As shown in Fig. 4b in Nat. Phys. **12** 52-56 (2016), we made a temperature gradient along the channel by applying much greater heat at the end of the channel than that induced by the pulsed pressure so as to investigate the thermoelectric effect. The result is that any voltage does not appear in this experiment, which indicates that thermal coupling between electrodes is established well in this setup.

The piezo effect caused by pressure is also eliminated, because we used quartz-glass capillary, not quartz capillary.

Following the referee's comment, we revised our manuscript.

Q.2

The meaning of the viscosity correction ξ in Equation 1 should be explained. Where does it refer to? The viscosity can cause a diffusion of vorticity away from the vortex cores and this can in principal influence the functionality of a SHDG.

A.2

Following the reviewer's comment, we added the explanation of ξ in the main text. The SHDG provides a new relaxation process to a liquid flow, which is not included in the conventional Navier-Stokes (NS) equation. Thus, as a back reaction of the SHDG, the viscosity in the NS equation is modified. The viscosity correction ξ represents the modification caused by the SHDG. The reference is described in eq. S31 in supplementary information of Nat. Phys. **12**, 52-56 (2016).

We are afraid that there is no vortex core in a laminar flow. The SHDG arises from the vorticity due to velocity distribution.

Q.3

Could the establishment of electric field, E(SHD), lead to parasitic effects? (for example, the occurrence of a Hall effect in their device)

A.3

If we apply an external magnetic field, a Hall effect is expected to occur. However, the external magnetic field leads the huge magnetohydrodynamic (MHD) effect. Thus, MHD effect becomes dominant and a Hall effect caused by the SHDG is masked by the MHD effect. Since another parasitic effect of SHDG may occur as a higher order effect of SHDG and may be smaller than the SHDG, we may not be able to detect it at present stage.

Q.4

Have the authors measured the transition from turbulent to laminar flow regime?

A.4

Following the reviewer's comments, we carried out additional experiments using a capillary of the diameter ϕ = 200 μm realizing a transition between the laminar and turbulent flow. In this measurement, *Re* can be varied from 200 to 2,210 with the pressure *P*.

The results are shown in Fig. R4.1. As shown in Fig. R4.1a, the *P* dependence of the mean velocity v changes from $\propto P$ in the low *P* region to $\propto \sqrt{P}$ in the high *P* region, indicating that the laminar flow in the low *P* region is converted into the turbulent flow in the high *P* region. In Fig. R4.1b, we show the v dependence of the voltage V_e. In the plot, V_e shows crossover from $\propto v$ in the low v region to $\propto v^2$ in the high v region. The intermediate region of v=0.5~1 ms^{-1} is the transition region form the laminal flow to the turbulent flow. This intermediate region coincides with the region of *P*=150~300 kPa in Fig. R4.1a.

In Fig. R4.1c, we show the *P* dependence of V_e. In the low *P* region, V_e is in good agreement with the black solid line, which is the theoretical calculation of the laminar SHDG using $\theta_{\mathrm{SHE}}\lambda^2\xi = 1.9\times 10^{-25}\ \mathrm{J\,s\,m^{-1}}$ estimated form the fit to the data in Fig. 3c in the main text.

In Fig. R4.1d, we show the $v_* r_0$ vs. $r_0^3 V_e L^{-1}$ plot so as to discuss the turbulent flow, where r_0 and L are the radius and the length of the capillary, respectively. v_* is the friction velocity (refer to p53 in Nat. Phys. **12** 52-56 (2016)). $r_0^3 V_e L^{-1}$ in the high $v_* r_0$ region, namely the high velocity region, is in good

agreement with the black solid curve, which is the theoretical calculation of the turbulent SHDG using $\theta_{\mathrm{SHE}}\lambda^2\xi = 5.9 \times 10^{-25}\ \mathrm{J\ s\ m^{-1}}$ estimated form the fit to the data in Fig. 2f in Nat. Phys. **12** 52-56 (2016).

Above results indicate that the low and high *Re* region is consistent with the SHDG theory of the laminar and turbulent flow, respectively.

We added these results, the corresponding paragraph and sentences to the main text.

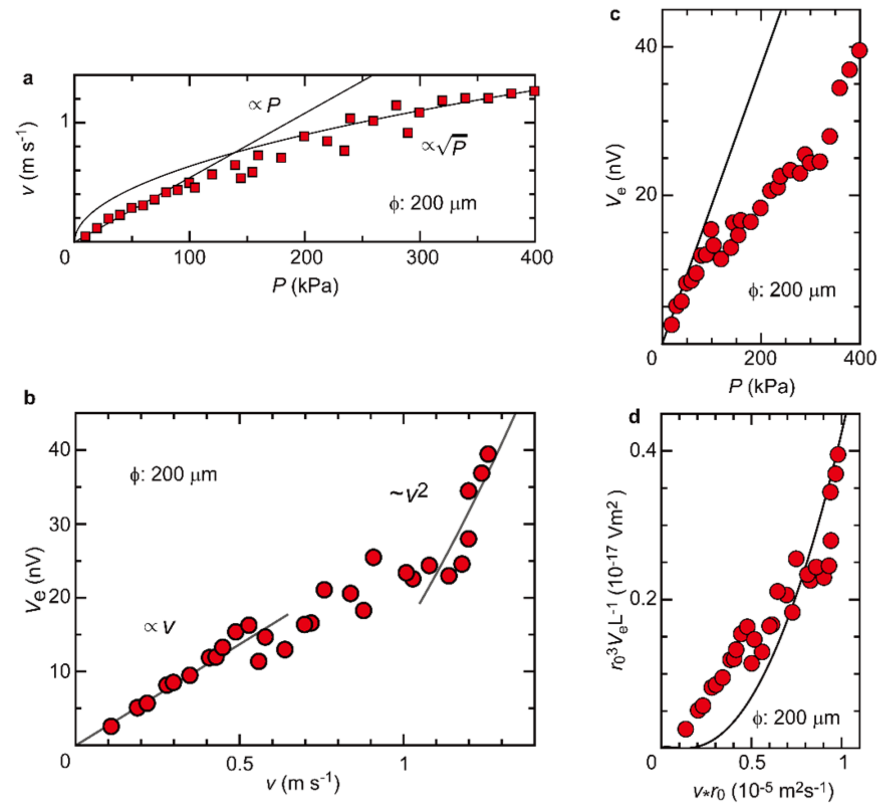

Fig. R4.1

Q.5

In spintronics values like θ(SHE), λ are of unique importance. How can they be envisaged in the case of liquid Hg in Equation 2? What is their order of magnitude?

A.5

In the case of a solid, *θ*(SHE) and *λ* have been measured using thin film with the thickness of about 10 nm. In the case of a liquid Hg, to form well-controlled thin film has not been realized. Thus, *θ*(SHE) and *λ* in liquid Hg have not been measured.

In general, θ(SHE) and λ depend on the spin-orbit interactions. Hg is a heavy element. Thus, θ(SHE) and λ in Hg are expected to be the same orders of heavy element like Pt. Typically, θ(SHE) and λ are about 0.01 and 10 nm, respectively.

Q.6-1

The conversion efficiency shown in Figure 4 is very small. Which factor establishes the maximum theoretical efficiency of a such spin hydrodynamic engine?

A.6-1

The theoretical upper limit of η is thought to be relevant to the two factors. The one is a spin-diffusion length and the other is a typical length of a flow. In terms of the spin current, when the typical length of the flow is larger than the spin-diffusion length, a spin current can be described by the spin-diffusion equation expressed in eq. (1) in Nat. Phys. **12** 52-56 (2016). Thus, SHDG is established. On the other hand, in terms of the fluid dynamics, our theory is based on the velocity distribution of a laminar flow described by the Hagen-Poiseuille (HP) equation. With decreasing the typical length of the flow, HP equation may be violated and the flow is described by the nanofluid dynamics before the typical length of the flow is comparable to the spin-diffusion length. Thus, the theoretical upper limit is determined by the typical length of the flow, which violates HP equation. In practical terms, when the roughness of the inner surface of the channel cannot be negligible, HP flow is violated.

Q.6-2

Although tube lengths of the mm size can be fabricated, are Reynold's numbers of the order of 10-6 realistic?

A.6-2

Re=10^{-6} can be realized by using microfluidic devices. As a matter of fact, we have made the microfluidic device with the hydraulic diameter of ~5 μm and the length of ~1 mm, and have flowed Hg with a velocity of ~0.1 μm/s for the purpose of another experiment. Re is estimated to be 10^{-6} order in this device. But we have not explored SHDG using this device yet, because we have not been able to overcome the difficulty of attaching the electrodes to this small device retaining the isothermal condition.

Q.6-3

How can this efficiency be compared to the power output of a magneto-hydrodynamic generator?

A.6-3

In general, output of MHD depends on an applied magnetic field. On the other hand, output of SHDG is irrelevant to a filed. Therefore, comparison of the efficiency between MHD and SHD depends on the experimental condition.

There are several advantages of SHDG as follows. The voltage V_e induced by the SHDG depends on the flow rate as shown in eq. (2) in the main text, thus the SHDG offer the electric measurement method of the flow rate without the disturbance caused by accessories like propeller and float in a channel. The SHDG also does not need accessories near a channel to measure the flow rate such as a pic-up coil, magnetic field and so on. The SHDG in the laminar flow is much more e□cient than that in the turbulent flow. Therefore, these advantages suggest that the SHDG may be applied to a miniaturized flow sensor for a liquid metal, which could be used, for example, as an active cooling system in a fast-breeder reactor, a semiconductor device and so on.

We added the corresponding paragraph in the main text.

REVIEWERS' COMMENTS:

Reviewer #1 (Remarks to the Author):

The authors have adequately addressed most of my comments and questions. I think the revised manuscript is suitable for publication in Nature Communications.

Reviewer #2 (Remarks to the Author):

The manuscript "Giant spin hydrodynamic generation in laminar flow", by R. Takahashi, et al., has been significantly improved by the authors, along the lines suggested by the referee reports. The most controversial issues have been addressed in a satisfactory way, and the answer are rather sound, if not convincing. While in the end a direct measure of the spin accumulation is highly desirable, I understand that, unfortunately, this is as yet beyond the reach of the authors' experimental setup.

As a final remark, I was wandering whether a figure reporting the efficiency η multiplied by r0^3, to remove the dependence on this parameter, might help the reader visualizing the smoothness of the laminar-to-turbulent crossover.

Once the author have considered this suggestion of mine, I think that their manuscript may be accepted for publication.

Reviewer #4 (Remarks to the Author):

The authors have satisfactory addressed all of my questions, performing new measurements and clarifying the findings. I have no further objections.

Reviewer #2 (Response to the Reviewer):

First of all, we are truly grateful to the reviewer for the examination of our manuscript and the consent to publish our paper in Nature Communications. The quality of the paper has been greatly improved by profiting from the reviewer's time and expertise. We appreciate all the comments and we present our response to the reviewer's final comment in the following, where the original comment is reproduced in italics.

The manuscript "Giant spin hydrodynamic generation in laminar flow", by R. Takahashi, et al., has been significantly improved by the authors, along the lines suggested by the referee reports. The most controversial issues have been addressed in a satisfactory way, and the answer are rather sound, if not convincing. While in the end a direct measure of the spin accumulation is highly desirable, I understand that, unfortunately, this is as yet beyond the reach of the authors' experimental setup.

Comment

As a final remark, I was wandering whether a figure reporting the efficiency η multiplied by r0^3, to remove the dependence on this parameter, might help the reader visualizing the smoothness of the laminar-to-turbulent crossover.

Answer

Thank you very much for your kind remark. Following the suggestion, we added the figure to display Re vs. $r_0^3\eta$ plot (Fig. 4e in the revised manuscript) and the corresponding sentences in the main text.

Once the author have considered this suggestion of mine, I think that their manuscript may be accepted for publication.
